# Immunological and Pathological Peculiarity of Severe Acute Respiratory Syndrome Coronavirus 2 Beta Variant

**DOI:** 10.1128/spectrum.02371-22

**Published:** 2022-08-25

**Authors:** Sunhee Lee, Gun Young Yoon, Su Jin Lee, Young-Chan Kwon, Hyun Woo Moon, Yu-Jin Kim, Haesoo Kim, Wooseong Lee, Gi Uk Jeong, Chonsaeng Kim, Kyun-Do Kim, Seong-Jun Kim, Dae-Gyun Ahn

**Affiliations:** a Center for Convergent Research of Emerging Virus Infection, Korea Research Institute of Chemical Technology, Daejeon, South Korea; b Department of Immunology, College of Medicine, Konkuk University, Chungju, South Korea; Fundacio irsiCaixa

**Keywords:** COVID-19, SARS-CoV-2, variant, cytokine, transcriptome

## Abstract

Diverse severe acute respiratory syndrome coronavirus 2 (SARS-CoV-2) variants have emerged since the beginning of the COVID-19 pandemic. We investigated the immunological and pathological peculiarity of the SARS-CoV-2 beta variant of concern (VoC) compared to the ancestral strain. Comparative analysis of phenotype and pathology revealed that the beta VoC induces slower disease progression and a prolonged presymptomatic period in the early stages of SARS-CoV-2 infection but ultimately causes sudden death in the late stages of infection in the K18-hACE2 mouse model. The beta VoC induced enhanced activation of CXCL1/2–CXCR2–NLRP3–IL-1β signal cascade accelerating neutrophil recruitment and lung pathology in beta variant-infected mice, as evidenced by multiple analyses of SARS-CoV-2-induced inflammatory cytokines and transcriptomes. CCL2 was one of the most highly secreted cytokines in the early stages of infection. Its blockade reduced virus-induced weight loss and delayed mortality. Our study provides a better understanding of the variant characteristics and need for treatment.

**IMPORTANCE** Since the outbreak of COVID-19, diverse SARS-CoV-2 variants have been identified. These variants have different infectivity and transmissibility from the ancestral strains. However, underlying molecular mechanisms have not yet been fully elucidated. In our study, the beta variant showed distinct pathological conditions and cytokine release kinetics from an ancestral strain in a mouse model. It was associated with higher neutrophil recruitment by increased levels of CXCL1/2, CXCR2, and interleukin 1β (IL-1β) at a later stage of viral infection. Our study will provide a better understanding of SARS-CoV-2 pathogenesis.

## INTRODUCTION

Coronavirus disease 2019 (COVID-19) is caused by severe acute respiratory syndrome coronavirus 2 (SARS-CoV-2), which emerged in Wuhan, China, in December 2019. The World Health Organization declared COVID-19 a global pandemic on 11 March 2020 (1). As of February 2022, more than 383 million cases and more than 5 million deaths have been reported worldwide (2). The symptoms of COVID-19 range from asymptomatic or mild to severe respiratory failure (3). Typical symptoms include fever, dry cough, shortness of breath, and fatigue. Acute respiratory distress syndrome, cardiovascular complications, thrombosis, and embolism occur in severe disease (4, 5). The excessive production of proinflammatory cytokines (“cytokine storm”) can lead to septic shock, tissue damage, and multiple-organ failure (6).

The global spread of SARS-CoV-2 and its natural mutations has led to the emergence of numerous variants around the world. Most mutations are likely to be neutral or slightly deleterious. However, some mutations affect the viral phenotype, associated with infectivity, disease severity, or host immunity (7). The World Health Organization has developed several nomenclature systems to determine the genetic lineage of SARS-CoV-2 and to identify variants of concern (VoCs) based on their public health significance (8, 9). As of late 2021, five VoCs had higher transmissibility, increased virulence, and reduced effectiveness of vaccines and therapeutics (9). These include the alpha variant (lineage B.1.1.7 clade GRY), which was identified in the United Kingdom in September 2020 (10); the beta variant (lineage B.1.351 clade GH [501Y.V2]), which was identified in South Africa in May 2020 (11); the gamma variant (lineage P.1 clade GR [501Y.V3]), which was identified in Brazil in November 2020 (12); the delta variant (lineage B.1.671.2 clade G [478K.V1]), which was identified in India in December 2020 (13); and the omicron variant (lineage B.1.1.529 clade GR [484A]), which was identified in Botswana in November 2020 (14). The emergence and rapid spread of VoCs have resulted in new waves of infection across the globe (15).

In South Korea, the SARS-CoV-2 strain originating in Wuhan, China, was the most prevalent strain during the first wave of the pandemic. Soon after the emergence of a variant with the D614G spike protein mutation in March 2020, other variants with additional mutations in the spike protein also emerged. The beta variant, which has D614G and N501Y mutations in the spike protein, was first identified in South Africa. It is associated with increased transmissibility and immune evasion. During the second wave of the pandemic, it rapidly became dominant globally, including in South Korea (11, 16, 17). Different outcomes were reported between the first two waves of the pandemic. Younger patients were infected during the second wave, with a high prevalence of the beta variant (18), which spread rapidly and was associated with more severe disease than earlier strains (11, 16, 19). Recent studies have revealed potential mechanisms underlying these observations. The D614G mutation and combined mutations in the spike protein have been shown to increase viral infectivity and enhance *in vivo* transmission in hamsters (20, 21). However, there is limited information on the pathogenesis and characteristics of VoCs.

We have recently studied the *in vitro* characteristics (infectivity, replication, and thermal stability) of several VoCs, including the alpha, beta, gamma, and delta variants (22). Among them, the beta variant had the highest infectivity and formed the largest plaques. Here, we describe the immunological and pathological aspects, and transcriptional changes, *in vivo* in K18-hACE2 mice infected with the beta variant compared to those occurring in infection with an early wild-type strain. Our findings are important for a better understanding of viral pathogenesis.

## RESULTS

### SARS-CoV-2 beta variant (B.1.351) had a longer presymptomatic period but a shorter time from the onset of symptoms to death in the K18-hACE2 mouse model.

**FIG 1:**
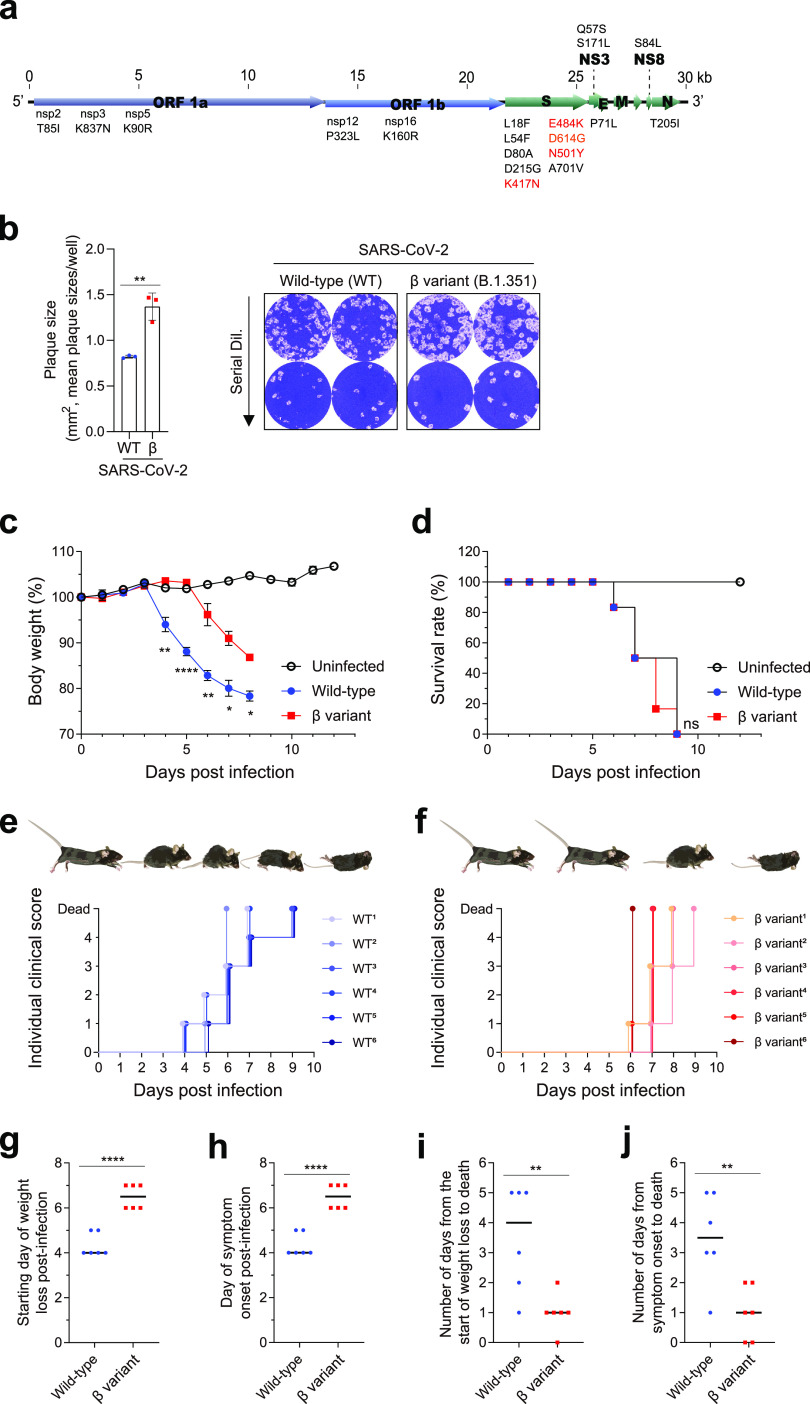
Comparison of *in vitro* infectivities and *in vivo* lethalities of severe acute respiratory syndrome coronavirus 2 (SARS-CoV-2) wild-type (WT) strain and beta (β) variant. (a) Schematic of SARS-CoV-2 genome. Mutations in the β variant and WT strain are shown. (b) Plaque sizes. Vero E6 cells were infected with serial dilutions (dil.) of the SARS-CoV-2 WT strain or β variant. Plaque areas were calculated. The mean plaque size per well is shown on the left. Representative plaques are shown on the right. Bars represent means ± SDs from three independent experiments. **, *P < *0.01 (Student’s *t* test). (c to f) Eight-week-old male K18-hACE2 mice (six mice per group) were infected intranasally with 2 × 10^3^ PFU of the WT strain or β variant of SARS-CoV-2. (c) Body weight. (d) Survival rate. (e and f) Individual clinical scores. Clinical status was scored according to the following grading scale: 0, healthy; 1, ruffled fur but active; 2, ruffled fur with reduced activity; 3, ruffled fur, inactive, and hunched; 4, imminent death; and 5, dead. Body weight symbols represent means ± SEM. *, *P < *0.05; **, *P < *0.01; ****, *P < *0.0001 (two-way analysis of variance with Tukey’s multiple-comparison test). (g) Starting day of weight loss postinfection. (h) Day of symptom onset postinfection. (i) Number of days from the start of weight loss to death. (j) Number of days from symptom onset to death. **, *P < *0.01; ****, *P < *0.0001 (Student’s *t* test).

To investigate the underlying pathogenesis associated with SARS-CoV-2 beta variant (B.1.351) infection, we compared the infectivities *in vitro* and lethalities *in vivo* of B.1.351 (hCoV-19/South Korea/KDCA0463/2020) (GISAID accession number EPI_ISL_762992) and an early wild-type strain (BetaCoV/Korea/KCDC03/2020) (GISAID accession number EPI_ISL_407193) isolated by the Korean Disease Control and Prevention Agency from travelers returning from Wuhan, China, and South Africa during the first and second waves, respectively (17, 23). Compared to the early wild-type strain, the beta variant has several mutations, including D614G and N501Y, which occur in the spike protein (Fig. 1a) and have been shown to increase infectivity and transmissibility (24, 25). The beta variant is also associated with 1.6-times-larger plaques (Fig. 1b), a measure of infectivity *in vitro* (26), consistent with a previous report (22).

We compared the pathogenesis and virus-induced immune responses of the beta variant and early wild-type strain in a transgenic mouse model. Although K18-hACE2 mice have different patterns of human angiotensin-converting enzyme 2 (ACE2) expression, driven by the human keratin 18 promoter, they are considered one of the most suitable animal models for characterizing SARS-CoV-2 variants, as they have similar pathological and immunological features of COVID-19-associated acute respiratory distress syndrome. There are also various scientific tools available for analysis and several previous reports in the literature (27–29).

K18-hACE2 mice (7 to 9 weeks old) were infected intranasally with 2 × 10^3^ PFU of SARS-CoV-2 to assess the outcomes of the wild-type strain and beta variant *in vivo*. At an infection dose of 2 × 10^3^ PFU, wild-type-infected mice showed typical phenotypic changes by respiratory pathogen infection (30). Consistent with a previous report (28), wild-type-infected mice gradually lost weight from 4 days postinfection (dpi), with a 20 to 25% reduction in body weight before death (Fig. 1c). Mice began to die at 6 dpi, and all mice died within 9 dpi (Fig. 1d).

Individual clinical scores were measured by monitoring each group of mice daily (Fig. 1e and f). Wild-type-infected mice gradually deteriorated from 1 to 4 dpi. They had reduced movement, ruffled fur, a hunched posture, and significant weight loss and died of severe illness (Fig. 1e). We initially anticipated a faster spread of the beta variant due to its higher infectivity *in vitro*. However, the beta variant had survival kinetics similar to those of the wild-type strain (Fig. 1d). Instead, beta variant-infected mice had outcomes different from those of wild-type-infected mice (Fig. 1c and f). Contrary to our initial expectations, beta variant-infected mice showed delayed weight loss (starting at 6 dpi), with less weight loss (10 to 15% reduction) before death than for wild-type-infected mice (Fig. 1c). Mice infected with a higher dose of the beta variant (1 × 10^4^ PFU) exhibited delayed weight loss (starting at 5 dpi), with significantly less weight loss (18.5% reduction) before death than for mice infected with 2 × 10^3^ PFU of the wild-type strain (see Fig. S1a and b in the supplemental material).

When individual mice were monitored, beta variant-infected mice died suddenly, with no or few symptoms (Fig. 1f). Weight loss or symptom onset began on average 6.5 dpi in beta variant-infected mice, which was delayed by 1.5 days compared to that in wild-type-infected mice (Fig. 1g and h). Symptoms developed more slowly in beta variant-infected mice, resulting in a longer presymptomatic period. Conversely, the mean time from weight loss or symptom onset to death in beta variant-infected mice was just 1 day, compared to 3 or 2.5 days, respectively, in wild-type-infected mice (Fig. 1i and j), suggesting that the beta variant is associated with a shorter disease duration. Although the SARS-CoV-2 wild-type strain and beta variant showed similar lethalities in the K18-hACE2 mouse model, different outcomes and physiological responses were observed, with gradual symptom onset but more rapid death after the onset of symptoms in beta variant-infected mice than in wild-type-infected mice.

### Comparison of viral load and lung pathology between SARS-CoV-2 wild-type- and beta variant-infected K18-hACE2 mice.

**FIG 2:**
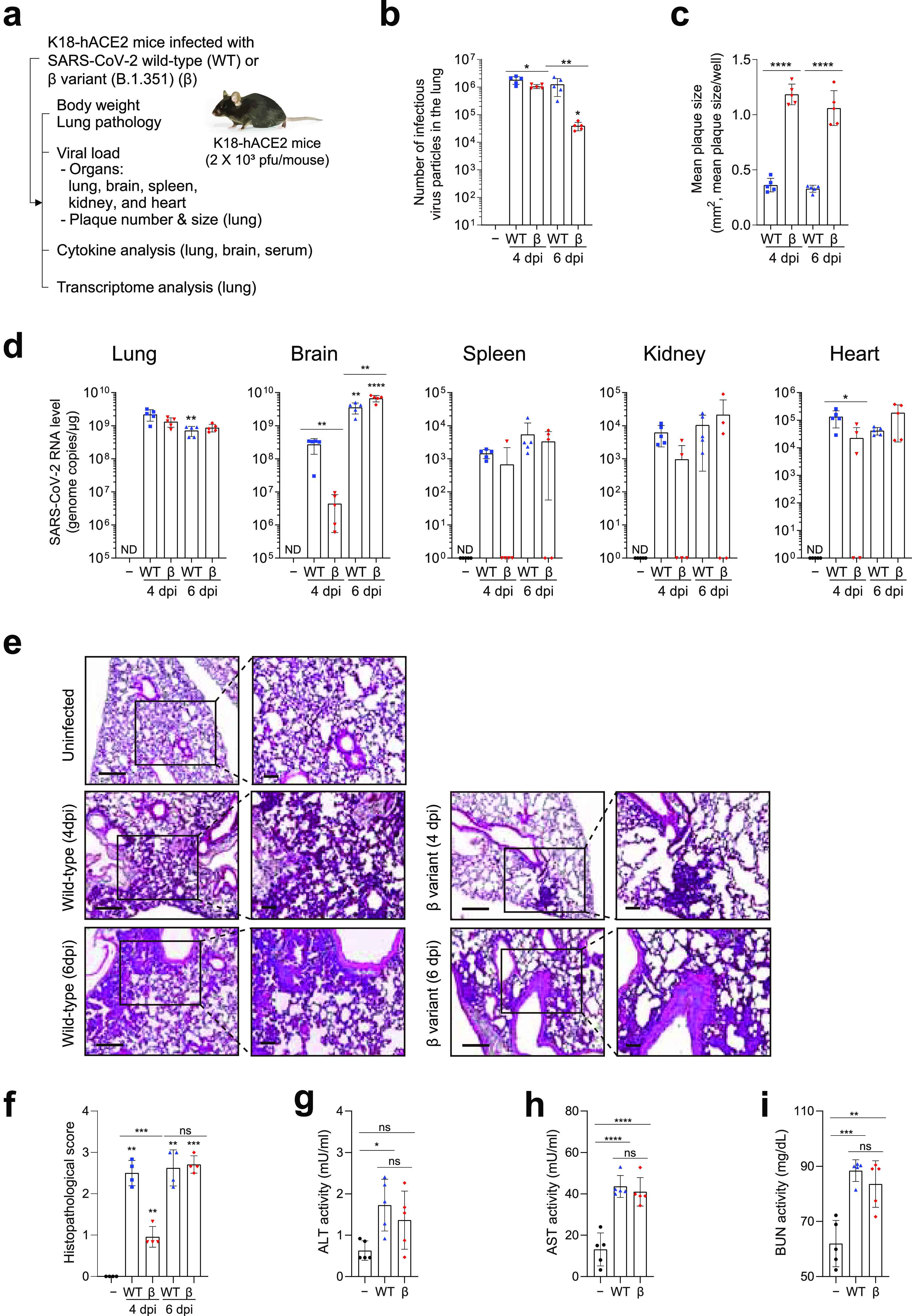
Viral load and pathology in various tissues of K18-hACE2 mice after SARS-CoV-2 infection. (a) Eight-week-old male K18-hACE2 mice (five mice per group) were infected intranasally with 2 × 10^3^ PFU of SARS-CoV-2 WT (blue symbols) or β variant (B.1.351) (red symbols). Body weight was monitored. Mice were sacrificed at 4 and 6 dpi. Serum and tissue samples were collected. Viral load, pathology, multiple-organ damage, and cytokine and transcriptome profiles were analyzed. Uninfected mice (−) (black symbols) were used as controls. (b) Number of infectious virus particles in the lung and (c) mean plaque size per well. Bars represent means ± SD from two independent experiments. *, *P < *0.05; **, *P < *0.01; ****, *P < *0.0001 (WT strain or β variant versus mock [two-way analysis of variance [ANOVA] with Sidak’s multiple-comparison test]; WT strain versus β variant [Student’s *t* test]). (d) Viral RNA levels by RT-qPCR in the lungs, brain, spleen, kidneys, and heart from two independent experiments. *, *P < *0.05; **, *P < *0.01; ***, *P < *0.001; ****, *P < *0.0001 (WT strain or β variant over time [two-way ANOVA with Bonferroni correction]; WT strain versus β variant [Student’s *t* test]). ND, not detected. (e and f) Histopathological examination of the lungs by hematoxylin and eosin staining (four mice per group). (e) Representative images of low-power (left) (scale bars, 200 μm) and high-power (right) magnification (scale bars, 100 μm). (f) Histopathological scores. Bars present means ± SD from four independent experiments. (g to i) Multiple-organ damage evaluated by measuring serum markers in WT- and β variant-infected mice at 6 dpi. (g) Alanine aminotransferase (ALT). (h) Aspartate aminotransferase (AST). (i) Blood urea nitrogen (BUN). *, *P < *0.05; **, *P < *0.01; ***, *P < *0.001; ****, *P < *0.0001. NS, not significant. (One-way ANOVA with Tukey’s multiple-comparison test was performed.)

Next, we examined the underlying pathogenesis of wild-type and SARS-CoV-2 beta variant (B.1.351) infection in K18-hACE2 mice by comparing the lung pathology, viral loads, and cytokine profiles (Fig. 2a). After inoculation with the wild-type or SARS-CoV-2 beta variant, mice were sacrificed at 4 and 6 dpi. Consistent with previous data, as shown in Fig. 1c, delayed weight loss was observed in beta variant-infected mice (Fig. S2). The number of infectious virus particles was evaluated in the lungs (the primary site of infection). High titers (>1.0 × 10^6^ PFU/g) of infectious virus particles were detected in both wild-type- and beta variant-infected mice at 4 dpi (Fig. 2b and Fig. S3a). While a high titer of infectious virus particles was maintained in the lungs of wild-type-infected mice, the virus titer (3.9 × 10^4^ PFU/g) in beta variant-infected mice was significantly reduced at 6 dpi. Plaque size was also measured. The plaque size of the virus in beta variant-infected mice was significantly larger than that in wild-type-infected mice at 4 and 6 dpi (Fig. 2c and Fig. S3b), consistent with previous data (Fig. 1b), suggesting that viral infectivity was not affected by *in vivo* inoculation. Viral RNA levels were assessed in the lungs, brain, spleen, kidneys, and heart. In the lungs, high viral RNA levels (7.1 × 10^8^ to 2.2 × 10^9^ genome copies/μg) were detected in both wild-type- and beta variant-infected mice at 4 and 6 dpi (Fig. 2d). In wild-type-infected mice, viral RNA was also detected in the brain, spleen, kidneys, and heart (secondary sites of infection) (31). This suggests that the virus had already spread to other organs at 4 dpi. In the brain, the virus titer (approximately 5 × 10^9^ genome copies/μg) further increased until 6 dpi. Conversely, in beta variant-infected mice, the viral load in all other organs was significantly lower at 4 dpi but rapidly reached similar levels by 6 dpi.

Lung histopathology was assessed by hematoxylin and eosin staining. No lesions or pathology were observed in uninfected mice. Immune cells accumulated predominantly in the perivascular regions of the lungs of wild-type- and beta variant-infected mice over the course of infection (Fig. 2e). Polymorphonuclear leukocyte infiltration involved a greater area of the lung, with focal accumulation in adjacent alveolar spaces and alveolar wall thickening. Immune cells were detected throughout the lung alveoli, along with interstitial edema, epithelial necrosis, pulmonary hemorrhage, and inflammation. These histopathological features were scored, and the scores at 6 dpi were comparable between the beta variant and wild-type strain (Fig. 2f). However, the lung pathology of beta variant-infected mice showed slower disease progress. Multiple-organ damage was evaluated by measuring markers of liver injury (alanine aminotransferase [ALT] and aspartate transaminase [AST]) (32) and kidney function (blood urea nitrogen [BUN]) (33). Wild-type- and beta variant-infected mice had higher levels of ALT, AST, and BUN at 6 dpi than did uninfected mice (Fig. 2g to i). The severities of multiple-organ damage were comparable between the beta variant and wild-type strain at 6 dpi.

Despite similar RNA viral levels and numbers of infectious virus particles in the early to middle stages of infection, lung pathology and viral spread to other organs progressed more slowly in beta variant-infected mice than in wild-type-infected mice. These findings correlated well with physiological changes, such as weight loss and delayed symptom onset. Comparable lung pathology and multiple-organ damage at 6 dpi suggest that virus-induced disease progressed rapidly in beta variant-infected mice in the later stages of infection. Therefore, we further investigated host immune responses during wild-type and SARS-CoV-2 beta variant (B.1.351) infection.

### Distinct cytokine kinetics during wild-type and SARS-CoV-2 beta variant infection.

**FIG 3:**
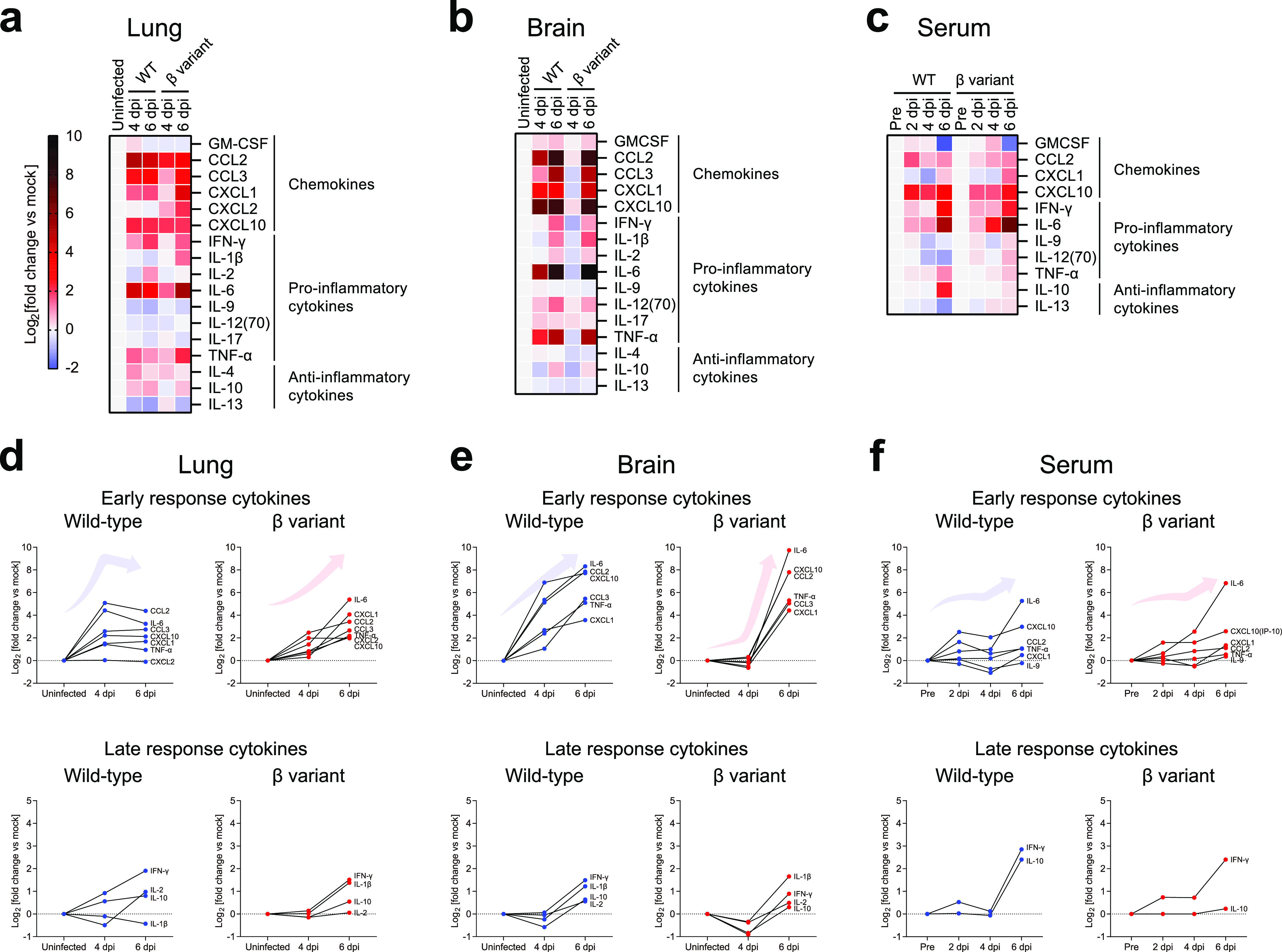
Chemokine and cytokine profiles after SARS-CoV-2 WT or β variant infection. Cytokine levels in the lungs (a and d), brain (b and e), and serum (c and f) from mice infected with SARS-CoV-2 WT (blue symbols) or β variant (red symbols) (five mice per group; two independent experiments) are shown. Uninfected mice (−) (black symbols) were used as controls. For serum analysis, samples collected before infection (pre) were used as controls. Sixteen cytokines were analyzed using a multiplex cytokine assay. The fold change of each cytokine was calculated compared to uninfected controls and plotted in heat maps (a to c) or on graphs (d to f). CXCL2 levels were additionally measured in the lungs by enzyme-linked immunosorbent assay. (a to c) Relative expression levels of chemokines, proinflammatory cytokines, and anti-inflammatory cytokines. (d to f) Relative expression levels of early-response (CCL2/3, CXCL1/10, IL-6, and TNF-α) and late-response (IL-1β/2/10 and IFN-γ) cytokines (see Fig. S4a to d for the actual expression level of each cytokine and statistics).

An excessive proinflammatory response to SARS-CoV-2 infection is the main driver of lung pathology and acute respiratory distress syndrome in patients with COVID-19 (6). We analyzed several key cytokines involved in the pathology and progression of COVID-19 in patients and animals infected with SARS-CoV-2 (5, 34, 35). Sixteen cytokines were analyzed in the lungs, brain, and serum of wild-type- and beta variant-infected mice using a multiplex cytokine assay (Fig. 3a to c and Fig. S4a to c). In the lungs, CXCL2 was additionally analyzed by enzyme-linked immunosorbent assay (Fig. 3a and Fig. S4d). A broad spectrum of chemokines and cytokines was excessively induced over time during viral infection in the lungs, brain, and serum of K18-hACE2 mice (Fig. 3a to c and Fig. S4a to c). Myeloid-associated chemokines and proinflammatory cytokines (CCL2/3, CXCL1/10, interleukin 6 [IL-6], and tumor necrosis factor alpha [TNF-α]) were induced earlier than T cell-associated and other proinflammatory cytokines (IL-1β/2/10 and gamma interferon [IFN-γ]) over the course of wild-type SARS-CoV-2 infection. Thus, we categorized these cytokines as early- or late-response cytokines (Fig. 3d to f). High levels of early-response cytokines (CCL2/3, CXCL1/10, IL-6, and TNF-α), granulocyte-macrophage colony-stimulating factor (GM-CSF), and IL-4 were detected in the lungs of wild-type-infected mice at 4 dpi. These levels were maintained or decreased slightly at 6 dpi (Fig. 3a and d and Fig. S4a). The levels of late-response cytokines increased gradually over time in the lungs of wild-type-infected mice. Conversely, in beta variant-infected mice, the levels of most early-response cytokines (CCL2/3, CXCL1, and TNF-α), except for CXCL10 and IL-6, remained significantly lower at 4 dpi, peaking at 6 dpi. The levels of GM-CSF and IL-2 remained low in beta variant-infected mice over the course of infection (Fig. 3a and Fig. S4a). Late-response cytokines were induced after 4 dpi. The late-response cytokine levels increased similarly to those in wild-type-infected mice at 6 dpi. The most profound differences in cytokine levels were in CXCL1/2 and IL-1β, which were excessively induced in the lungs of beta variant-infected mice but not of wild-type-infected mice, at 6 dpi (Fig. 3a and d and Fig. S4a and d). Interestingly, low or no induction of CXCL1/2 and IL-1β was observed by another group in the lungs of wild-type-infected mice (27). The induction of these cytokines likely led to a reduction in the number of infectious virus particles in the lungs of beta variant-infected mice (Fig. 2b).

In the brains of wild-type-infected mice, the levels of early-response cytokines (GM-CSF and IL-12 [p70]) began to increase at 4 dpi, peaking at 6 dpi (Fig. 3b and e and Fig. S4b). The levels of late-response cytokines increased after 4 dpi. Compared to the cytokine profile in the lungs of wild-type-infected mice, there was a delay in the release of cytokines in the brain, which correlated with the spread of the virus from the lungs to other organs (Fig. 2d), consistent with a previous report (27). In the brains of beta variant-infected mice, the levels of early- and late-response cytokines remained low at 4 dpi but increased substantially at 6 dpi (Fig. 3e). Compared to wild-type-infected mice, beta variant-infected mice had slightly higher levels of CXCL1 and IL-1β and considerably higher levels of IL-6 at 6 dpi.

In serum, there was a lower induction of CCL2 and CXCL10 during the early stages of infection in beta variant-infected mice (Fig. 3c and Fig. S4c). While CCL2 and CXCL10 were robustly induced in wild-type-infected mice at 2 dpi, the levels of these cytokines in beta variant-infected mice gradually increased over time, with significantly low levels at 2 dpi. IL-10 was induced in wild-type-infected mice, but not in beta variant-infected mice, at 6 dpi (Fig. 3c).

Beta variant-infected mice were more burdened by excessive cytokine release at a relatively late stage of infection than were wild-type-infected mice. Cytokine kinetics were highly correlated with viral load and physiological changes, such as weight loss and clinical score. This may explain the prolonged presymptomatic period and sudden death associated with the beta variant compared to that with the early wild-type strain.

### Enhanced inflammation activation via the CXCL1/2–CXCR2–NLRP3–IL-1β axis in the lungs of SARS-CoV-2 beta variant (B.1.351)-infected mice.

**FIG 4:**
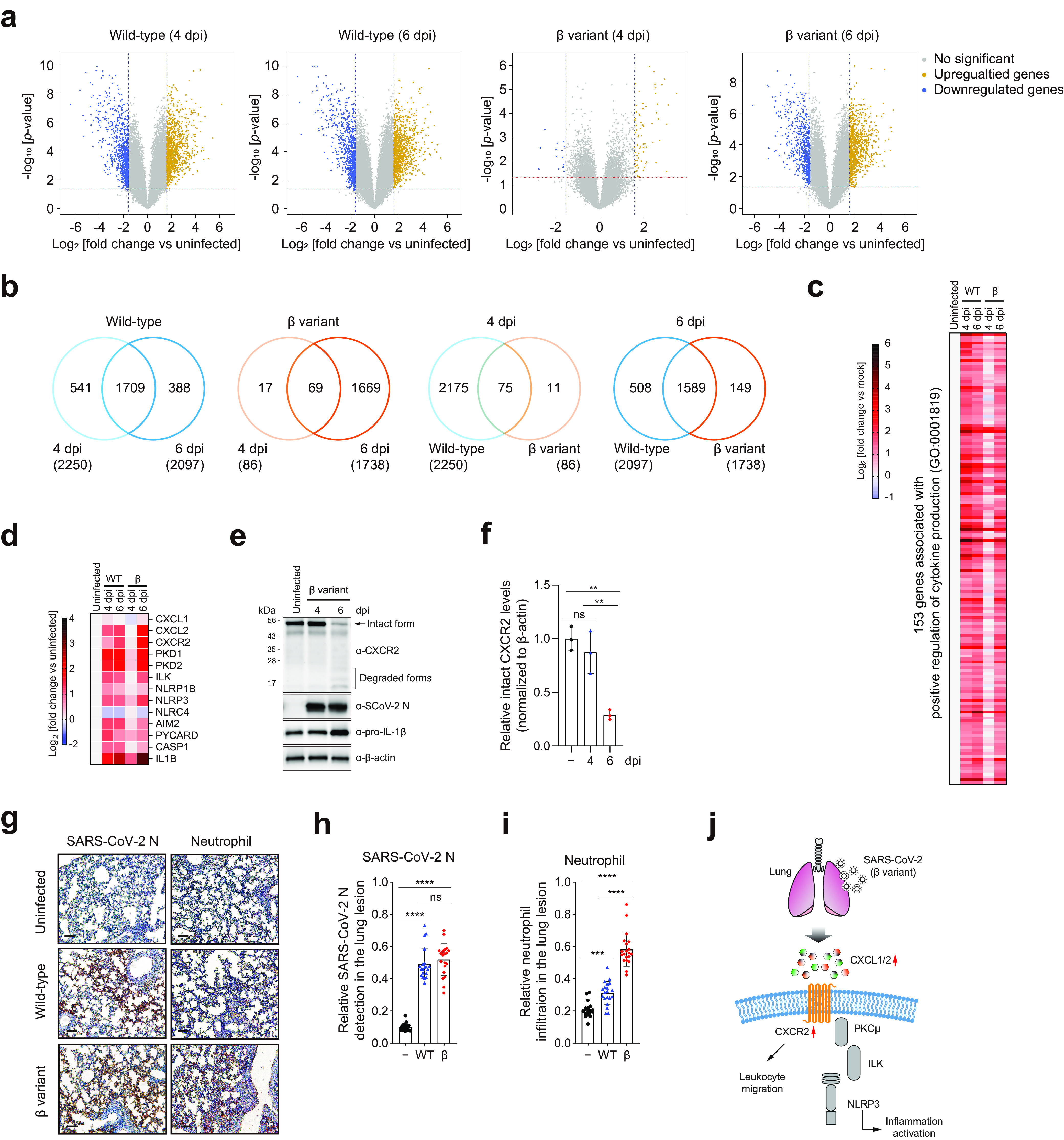
Transcriptome profiling of the lungs of K18-hACE2 mice infected with SARS-CoV-2 WT strain or β variant. (a to d) Whole-transcriptome profiling of lung homogenates was performed by microarray analysis (five mice per group). Uninfected mice were used as controls. (a) Volcano plots of differentially expressed genes (DEGs) in the lungs of virus-infected mice versus uninfected mice at 4 and 6 dpi. Up- and downregulated genes are represented by dark yellow and blue dots, respectively (fold change ≥ 3 [blue vertical dashed lines]; *P < *0.05 [red horizontal dashed lines]). (b) Venn diagrams showing the overlap of DEGs between different time points or virus strains compared to uninfected controls. Numbers in parentheses indicate the total number of DEGs. (c) Heat map of significantly upregulated genes annotated with the Gene Ontology (GO) term “positive regulation of cytokine production” (GO:0001819). (d) Heat map of genes involved in inflammasome activation. (e and f) Western blot analysis of the indicated proteins in the lungs of K18-hACE2 mice infected with the β variant of SARS-CoV-2. (e) Representative images (three mice per group). The full-length intact form of CXCR2 is indicated by the arrow. (f) The ratio of intact CXCR2 to β-actin was determined by densitometric analysis. **, *P < *0.01 (one-way analysis of variance with Tukey’s multiple-comparison test). (g to i) Neutrophil infiltration into lung lesions. Immunohistochemistry was performed on lung sections from K18-hACE2 mice infected with the WT strain or β variant of SARS-CoV-2 at 6 dpi using an anti-SARS-CoV-2 nucleocapsid (N) protein antibody (left) and a neutrophil marker (right). (g) Representative images (five mice per group) (scale bars, 100 μm). (h and i) SARS-CoV-2 N protein expression (h) and neutrophil infiltration (i) relative to the total lesion area for each of 20 acquired images per group. ***, *P < *0.001; ****, *P < *0.0001 (one-way analysis of variance with Tukey’s multiple-comparison test). (j) Schematic of the NLRP3 inflammasome signaling pathway.

To investigate a broad spectrum of immunological responses and to determine the underlying mechanisms of differentially expressed cytokines, such as CXCL1/2 and IL-1β, we analyzed the whole transcriptomes of lung homogenates from wild-type- and beta variant-infected mice at 4 and 6 dpi. Diverse genes were differentially expressed in virus-infected mice compared to uninfected mice (Fig. 4a). In wild-type-infected mice, >2,000 genes were differentially expressed at 4 and 6 dpi, with 1,709 genes in common (Fig. 4b). In contrast, in beta variant-infected mice, only 86 genes were differentially expressed at 4 dpi, while 1,738 genes were differentially expressed at 6 dpi. Most differentially expressed genes in beta variant-infected mice were shared with wild-type-infected mice at 6 dpi. Gene Ontology (GO) analysis of “biological process” terms revealed that most of the genes enriched in virus-infected mice were associated with immunological processes, such as “response to IFN-β,” “defense response to virus,” “cytokine-mediated signaling pathway,” and “cytokine production” (Fig. S5a to d). “Positive regulation of cytokine production” (GO:0001819) and “cytokine-mediated signaling pathway” (GO:0019221) were the highest-ranked terms in wild-type-infected mice at 4 and 6 dpi and in beta variant-infected mice at 6 dpi. “Regulation of innate immune response” (GO:0045088) and “response to IFN-β” (GO:0035456) were highly ranked in beta variant-infected mice at 4 dpi, suggesting that infected mice exhibit early stages of virus-induced immune response at this point. “Positive regulation of cytokine production” (GO:0001819) included 153 genes that were significantly upregulated during infection (Fig. 4c). The overall expression patterns of upregulated genes in wild-type- and beta variant-infected mice were remarkably similar and correlated well with the kinetics of cytokine release.

A multiplex cytokine assay revealed higher levels of cytokines such as CXCL1/2 and IL-1β in the lungs of beta variant-infected mice (Fig. 3 and Fig. S4). Therefore, we examined gene expression changes of *Cxcl1/2*, *Cxcr2*, *Pkd1* (also known as *Pkcμ*), *Nlrp3*, and *Il-1β* in inflammasome-mediated signaling pathways (Fig. 4d). These genes were highly expressed during viral infection. Notably, *Cxcl2*, *Cxcr2*, and *Il-1β* expression increased significantly in beta variant-infected mice at 6 dpi compared to that at 4 dpi. To some extent, expression levels were higher than those in wild-type-infected mice at 6 dpi (Fig. 4d and Fig. S6).

CXCR2 is a G-protein-coupled receptor. CXCL1/2 binding mediates neutrophil migration and NLRP3-dependent activation of inflammation (36–38). When activated by ligand binding, CXCR2 initiates signal transduction and undergoes clathrin-mediated endocytosis, followed by lysosomal degradation (39–41). Thus, we assessed CXCR2 status in the lungs of beta variant-infected mice over the course of infection. Full-length intact CXCR2 was detected in uninfected mice using antibodies against its C terminus (Fig. 4e). Even in the presence of viral proteins at 4 dpi, CXCR2 was in the intact form due to low levels of CXCL1/2 and IL-1β at this point (Fig. 3a and Fig. 4e). In contrast, a significant reduction in the intact form of CXCR2, with a concomitant increase in the degraded form and a notable increase in IL-1β, was observed in virus-infected mice at 6 dpi, suggesting the activation of CXCR2 during SARS-CoV-2 infection (Fig. 4e and f). As CXCR2 plays a crucial role in the recruitment of neutrophils into the lung (38), we also analyzed by immunohistochemistry, using an anti-SARS-CoV-2 nucleocapsid protein antibody and a neutrophil marker, neutrophil migration into the lung lesions of virus-infected mice. While the SARS-CoV-2 nucleocapsid protein was not detected in uninfected mice, nucleocapsid protein expression was localized primarily to alveolar epithelial cells in virus-infected mice at 6 dpi (Fig. 4g). Similar amounts of nucleocapsid protein were found in the lung lesions of wild-type- and beta variant-infected mice at 6 dpi (Fig. 4h). Neutrophil infiltration around the lungs was evident in virus-infected mice but not in uninfected mice (Fig. 4g). Relative neutrophil infiltration was higher in beta variant-infected mice (Fig. 4i), suggesting that more neutrophils were recruited to the lung lesions in beta variant-infected mice than in wild-type-infected mice at 6 dpi. This suggests that neutrophils are rapidly recruited by high levels of CXCL1/2 and CXCR2 in beta variant-infected mice despite delayed cytokine release compared to that in wild-type-infected mice. GO analysis also supports this conclusion. “Leukocyte migration” (GO:0050900), “leukocyte chemotaxis” (GO:0030595), and “myeloid leukocyte migration” (GO:0097529) were ranked in the top 20 terms in beta variant-infected mice only at 6 dpi (Fig. S5d). Collectively, these findings demonstrated that enhanced inflammation activation via the CXCL1/2–CXCR2–NLRP3–IL-1β axis and neutrophil recruitment occur in the lungs of beta variant-infected mice in the later stages of infection (Fig. 4j).

### Delayed lethality of SARS-CoV-2 beta variant (B.1.351) by CCL2 blockade.

**FIG 5:**
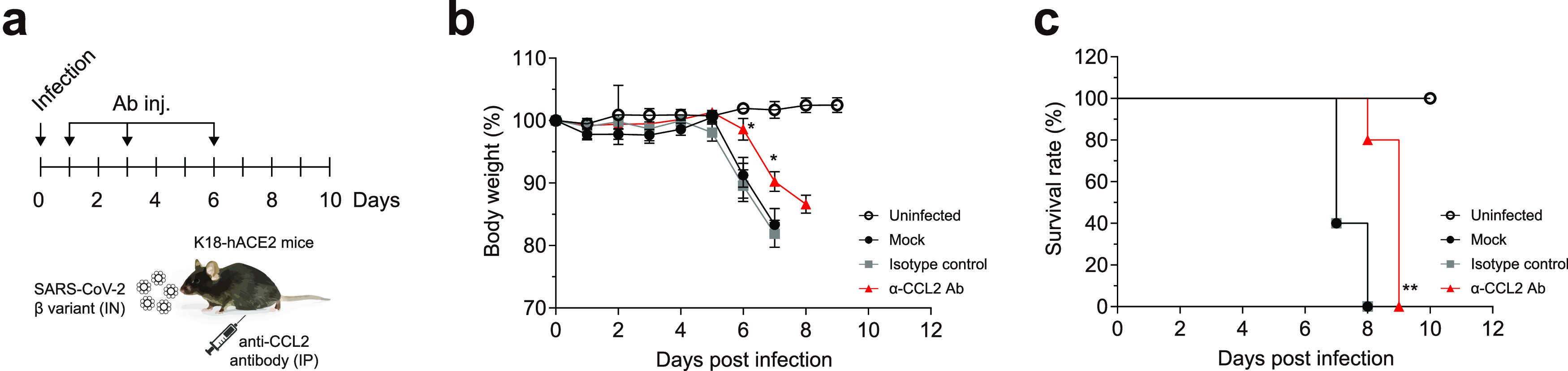
Delayed virus-induced death of K18-hACE2 mice by proinflammatory cytokine blockade. (a) Eight-week-old male K18-hACE2 mice (five mice per group) were infected intranasally (IN) with 2 × 10^3^ PFU of SARS-CoV-2 β variant. Blocking antibodies (Ab) against CCL2 (5 mg/kg) were administered intraperitoneally (IP) at 1, 3, and 6 dpi. An isotype Ab was used as a negative control. (b) Body weight. (c) Survival rate. Body weight symbols represent means ± SEM. *, *P < *0.05; **, *P < *0.01 (two-way analysis of variance with Tukey’s multiple-comparison test [body weight]; log-rank [Mantel-Cox] test [survival rate]).

Cytokine blockade can be useful in preventing inflammation and delaying disease progression. In this study, CCL2 was found to be the earliest and most strongly released cytokine during SARS-CoV-2 infection. Therefore, we tested whether blocking this cytokine could delay virus-induced death in beta variant-infected mice. An isotype antibody was used as a negative control. Mock, isotype, and anti-CCL2 antibodies were administered at 1, 3, and 6 dpi (Fig. 5a). Mock- and isotype control-treated mice showed significant weight loss and died at 8 dpi (Fig. 5b and c). In contrast, anti-CCL2 antibody reduced weight loss and delayed death. Thus, CCL2 blockade may help to delay or reduce the “cytokine storm” in patients with COVID-19.

## DISCUSSION

We compared the immunopathological patterns and transcriptome changes during infection between the SARS-CoV-2 beta variant (B.1.351), which dominated during the second wave of the pandemic, and the ancestral wild-type strain originating from Wuhan, China. The pathophysiological characteristics of the beta variant were that it caused rapid deterioration and death. K18-hACE2 mice exhibited few or no symptoms during the beta variant infection despite similar lethality and lung pathology before death. The beta variant spread unexpectedly slowly from the lungs (the primary site of infection) to other organs (secondary sites of infection). This was accompanied by delayed cytokine release compared to that in wild-type-infected mice, resulting in a prolonged presymptomatic period. Viral transmission during the presymptomatic period is crucial for the spread of COVID-19. Several cohort studies and meta-analyses have shown that presymptomatic transmission contributed substantially (37 to 75%) to the spread of COVID-19 during the pandemic (42–46). Presymptomatic, asymptomatic, and mildly symptomatic patients can be nearly as infectious as hospitalized patients (47). The D614G mutation has also been shown to increase virus propagation power in Syrian hamsters (21). Therefore, we postulated that VoCs may have evolved to have different presymptomatic periods, which affect spread, dominance, and infectivity. It is highly likely that individuals infected with SARS-CoV-2 VoCs who have mild symptoms or a long presymptomatic phase will transmit the virus without being aware that they are infected. Otherwise, they would be quarantined or hospitalized to limit their social contact.

The excessive production of proinflammatory cytokines (cytokine storm) is the main driver of disease severity and progression and multiple-organ failure in patients with COVID-19 (3, 48). In particular, the levels of serum and bronchoalveolar lavage fluid proinflammatory chemokines and cytokines (CCL2/3, CXCL10, IL-6/10, IFN-γ, and TNF-α) are highly correlated with disease severity and progression in patients with COVID-19 (5, 34, 35). High levels of anti-inflammatory cytokines (IL-4/10/13), secreted by T helper 2 cells (49), have also been detected in patients with severe COVID-19 (5). The expression profiles of these cytokines, determined by multiplex cytokine assay and whole-transcriptome analysis in the K18-hACE2 mouse model, were highly correlated with those of patients with COVID-19. Cytokine profiles and physiological changes induced by the wild-type strain were consistent with those by earlier strains of SARS-CoV-2 (27–29). However, in beta variant-infected mice, there was a characteristic shift in chemokine and cytokine release toward the later stages of infection, with a marked increase and greater inflammatory burden. This involves physiological changes, such as delayed symptom onset and sudden death. Interestingly, a similarly delayed, but severe, cytokine storm was observed during SARS-CoV-2 infection in aged Chinese rhesus macaques (50). Young macaques had more active immune responses with subsequent improvement in respiratory function, whereas aged macaques, which had less active immune responses, had moderate inflammation in the early stages of infection but suffered a cytokine storm in the later stages of infection (50). Immunological responses to the beta variant resembled those of aged animal models. It is possible that the beta variant may have evolved to trigger low or moderate inflammation in the early stages of infection to evade host immunity in younger individuals. As there were more infections among younger people during the second wave of the pandemic (18), this hypothesis requires further investigation.

Cytokines IL-1β and IL-6 act as key mediators of hyperinflammation in patients with COVID-19 (51). Initially, we and other groups observed no or low levels of IL-1β in the lungs of K18-hACE2 mice over the course of infection with the wild-type strain (2.4 × 10^4^ to 1 × 10^5^ PFU) (27, 29, 52). IL-1β production was only observed in the lungs of K18-hACE2 mice infected with a high (10^5^), and not a low (10^4^; 50% tissue culture infective dose), dose of the wild-type strain (28). Interestingly, we found that CXCL1/2 and IL-1β were more abundant in the lungs of beta variant-infected mice. We also demonstrated that enhanced inflammation activation via the CXCL1/2–CXCR2–NLRP3–IL-1β axis and neutrophil recruitment occurs in the lungs of beta variant-infected mice in the later stages of infection. Murine CXCL1 (KC) and CXCL2 (MIP-2) are functional homologs of human CXCL1 (GRO-α), CXCL2 (GRO-β), CXCL3 (GRO-γ), and CXCL8 (IL-8) (53–55). CXCL1/2 are major neutrophil chemoattractants that transduce signals by binding to the receptor, CXCR2 (56–58), which mediates neutrophil recruitment and migration, as well as inflammation, in acute respiratory distress syndrome and acute lung injury (38, 54–56, 59, 60). Neutrophil recruitment is highly correlated with the severity of COVID-19. Higher neutrophil counts were observed in critically versus noncritically ill patients and in those who died suddenly after admission versus those who did not (61, 62). These observations are consistent with our findings supporting enhanced neutrophil recruitment with the rapid development of lung pathology in beta variant-infected mice.

The CCL2-CCR2 axis is another major driver of inflammation-mediated monocyte recruitment (63, 64). Thus, it is an important therapeutic target for suppressing hyperinflammation in patients with COVID-19 (65). Our multiplex cytokine assay and transcriptome analysis showed that CCL2 was one of the most highly secreted cytokines during SARS-CoV-2 infection. CCL2 blockade delayed virus-induced weight loss and death in the K18-hACE2 mouse model, providing a therapeutic opportunity to protect against septic shock and improve mortality. This could be a beneficial strategy for the treatment of patients with presymptomatic or mild disease. A recent study reported that SARS-CoV-2-induced inflammatory cytokine levels were reduced in CCR2^–/–^ mice compared to C57BL/6 wild-type mice, due to limited recruitment of monocytes and monocyte-derived cells into the lungs (66). Combined treatment with antiviral agents and anti-inflammatory inhibitors may provide promising therapeutic strategies for the treatment of patients with severe COVID-19.

Our study is limited in that most experiments were performed in the K18-hACE2 mouse model. Further studies are needed to elucidate the precise mechanisms by which viral mutations are associated with these observations. An approach using reverse genetic systems (e.g., SARS-CoV-2 infectious clones harboring mutations) should reveal the underlying mechanisms. Nevertheless, as the cytokine profiles and lung pathology in K18-hACE2 mice closely resemble those in patients with COVID-19, the unique characteristics of VoCs should be considered in the diagnosis, prognosis, and treatment of COVID-19. Our comprehensive characterization of the beta variant (B.1.351) provides insights into the pathogenesis of VoCs and their evolutionary trajectories.

## MATERIALS AND METHODS

### Viruses and cell culture.

SARS-CoV-2 wild-type strain (BetaCoV/Korea/KCDC03/2020) (GenBank accession number MW466791; GISAID accession number EPI_ISL_407193) and beta variant (B.1.351) (hCoV-19/South Korea/KDCA0463/2020) (GISAID accession number EPI_ISL_762992) were obtained from the National Culture Collection for Pathogens (NCCP; numbers 43326 and 43382, respectively) of the Korea Disease Control and Prevention Agency. Viruses were propagated in Vero E6 cells (ATCC; CCL-1586) (17) maintained in Eagle’s minimum essential medium and RPMI 1640 medium supplemented with 10% fetal bovine serum, 100 U/mL of penicillin, and 100 μg/mL of streptomycin under standard culture conditions (5% carbon dioxide at 37°C). Genetic information was obtained via the Global Initiative on Sharing All Influenza Data (GISAID). The sequences were previously confirmed by our group (22). All experiments were conducted in a biosafety level 3 laboratory at the Korea Research Institute of Chemical Technology.

### Plaque formation assay.

Viruses and lung homogenates from K18-hACE2 mice infected with SARS-CoV-2 wild-type strain or beta variant at 4 and 6 dpi were serially diluted in Eagle’s minimum essential medium supplemented with 2% fetal bovine serum. The cell culture medium was removed from Vero E6 cells (1 × 10^5^ per 24-well plate) before the inoculum was transferred onto triplicate cell monolayers. Cells were incubated at 37°C for 1 h. The inoculum was discarded before the infected cells were overlaid with 0.9% carboxymethyl cellulose in minimum essential medium. Cells were incubated at 37°C for 4 days before being fixed and stained with 0.05% crystal violet in 1% formaldehyde. Plaque counts and size evaluations were performed using an ImmunoSpot analyzer (Cellular Technology Ltd.).

### Animal models.

All experiments were conducted in a biosafety level 3 facility. Animal protocols were reviewed and approved by the Institutional Animal Care and Use Committee of the Korea Research Institute of Chemical Technology (permit no. 2021-8A-04-01, protocol no. 8A-M6). Male K18-hACE2 (no. 034860) [B6.Cg-Tg(K18-ACE2)2Prlmn/J] mice were purchased from the Jackson Laboratory. Mice were randomly divided into groups before infection. For SARS-CoV-2 infection, mice were lightly euthanized with isoflurane and injected intranasally with 50 μL of saline containing 2 × 10^3^ PFU of SARS-CoV-2 wild-type strain or beta variant. Body weight, clinical score, and survival were monitored daily after infection. The initial day of weight loss was defined as the first day with a >5% reduction in initial body weight. Clinical status was scored according to the following grading scale (30): 0, healthy; 1, ruffled fur but active; 2, ruffled fur with reduced activity; 3, ruffled fur, inactive, and hunched; 4, imminent death; and 5, dead. Mice were sacrificed at 4 and 6 dpi for analysis of viral load and histopathological analysis. For cytokine blockade, beta variant-infected mice were injected intraperitoneally with 5 mg/kg of body weight of CCL2/JE/MCP-1 antibody (AB-479-NA) and normal goat IgG control (AB-108-C) (R&D Systems) at 1, 3, and 6 dpi. Efforts were made to minimize animal suffering.

### Tissue isolation.

Uninfected and SARS-CoV-2 (wild-type strain or beta variant)-infected mice were anesthetized with isoflurane. The lungs, brain, spleen, kidneys, and heart were perfused with 10 mL of phosphate-buffered saline (PBS; Gibco) to remove blood. Tissue sections were homogenized in cold PBS using a FastPrep-24 homogenizer (MP Biomedicals) for three cycles of 15 s on and 20 s off. Debris was removed by brief centrifugation.

### RT-qPCR.

Total RNA was isolated from homogenized tissues using a commercially available kit (Maxwell RSC simplyRNA tissue kit) (Promega). Quantitative reverse-transcription PCR (RT-qPCR) (QuantStudio 3; Applied Biosystems) was performed using one-step Prime Script III RT-qPCR mix (TaKaRa, Japan). SARS-CoV-2 RNA levels were quantified using a 2019-nCoV-N1 probe (no. 10006770) (primer sequence: 5′-6-carboxyfluorescein (FAM)-ACAATTTGCCCCCAGCGCTTCAG-BHQ1; Integrated DNA Technologies) targeting the SARS-CoV-2 nucleocapsid protein.

### Histology and immunohistochemistry.

After transcardial perfusion with PBS, the lungs of SARS-CoV-2-infected K18-hACE2 mice were dissected and fixed in 10% neutral buffered formalin at room temperature for 2 to 5 days. Tissue sections (4 μm thick) were stained with hematoxylin and eosin for light microscopic examination. Immunohistochemistry was performed as previously described (67), with some modifications. Sections were deparaffinized in an oven at 65°C and rehydrated sequentially with 100%, 90%, 80%, and 70% xylene. Sections were then rinsed three times with PBS. For antigen retrieval, sections were microwaved in 10 mM citrate buffer for 30 min. Sections were cooled to room temperature for a minimum of 20 min and incubated with 3% hydrogen peroxide. Nonspecific background staining was blocked with goat serum for 1 h at room temperature. Sections were incubated at 4°C overnight with SARS-CoV/SARS-CoV-2 nucleocapsid (no. 40143-R001; Sino Biological) or anti-neutrophil marker antibody (no. sc-71674; Cell Signaling Technology). The next day, sections were incubated with biotinylated goat anti-rabbit or goat anti-rat IgG for 1 h, followed by ABC/HRP complex (Vectastain ABC kit, peroxidase rat IgG PK-4004, and peroxidase rabbit IgG PK-4001; Vector Laboratories). Staining was visualized using 3’3-diaminobenzidine (DAB) (SK-4100; Vector Laboratories) for 3 min. Sections were counterstained with hematoxylin (H-3404-100) (QS counterstain). Images were acquired using an Olympus DP70 camera mounted to an Olympus BX60 microscope (Olympus) and CellF imaging software (Olympus). Relative SARS-CoV-2 infection and neutrophil-infiltrated areas were quantified as integrated optical density using ImageJ software (National Institutes of Health) (68).

### Histopathological scoring criteria.

Pathological changes in the lungs were quantified as previously described (69), with some modifications. Interstitial edema, epithelial necrosis, pulmonary hemorrhage, and degree of inflammation were evaluated by measuring the area of perivascular infiltration with respect to the total lung section area. An average of three sections per group were evaluated for each item, graded as follows: 0, none; 1, low; 2, moderate; 3, high; and 4, very high.

### Multiplex cytokine assay.

Serum was prepared from blood centrifuged at 1,500 rpm for 15 min. Tissue debris and cells in lung and brain homogenates were removed by centrifugation. Serum and supernatant of lung and brain homogenates were treated with 1% Triton X-100 to inactivate SARS-CoV-2 (29). Cytokines and chemokines were analyzed using the MILLIPLEX MAP mouse cytokine/chemokine magnetic bead panel (MCYTOMAG-70K-PMX; Merck Millipore), according to the manufacturer’s instructions.

### Whole-transcriptome analysis.

Whole-transcriptome analysis was performed using the Affymetrix GeneChip Mouse Gene 2.0 ST array (no. 902463) and GeneChip WT Pico kit (no. 902623) (Thermo Fisher Scientific) according to the manufacturer’s instructions. The raw data were summarized and normalized using the Robust Multi-Average method in Affymetrix Power Tools, followed by differential expression analysis. Statistical significance was determined using an independent *t* test and fold change. The null hypothesis was that no difference exists between the groups. The false-discovery rate was controlled by adjusting the *P* value using the Benjamini-Hochberg algorithm. Gene enrichment and functional annotation analysis for the significant probe list were performed using GO (http://geneontology.org). All data analyses and visualization were performed in R 3.3.2 (www.r-project.org).

### Biochemical analysis.

Multiple-organ damage was evaluated by determining the levels of serum AST and ALT (markers of liver injury) and BUN (a marker of kidney injury). AST, ALT, and BUN levels were measured using an AST activity assay kit (no. ab105135), ALT assay kit (no. ab105134), and urea assay kit (no. ab83362) (Abcam), respectively, according to the manufacturer’s instructions. For Western blot analysis, cellular or viral proteins were detected in lung homogenates using anti-SARS-CoV-2 nucleocapsid protein (no. 40143-R001; Sino Biological), anti-β-actin (no. sc-47778; Santa Cruz Biotechnology), anti-CXCR2 (no. ab217314; Abcam), and anti-IL-1β (no. 12242; Cell Signaling Technology) antibodies. Blots were incubated with the appropriate horseradish peroxidase-conjugated secondary antibody (Cell Signaling Technology). Membranes were developed with enhanced chemiluminescence solution (PerkinElmer) and imaged using an ImageQuant Las 4000 system (GE Healthcare). Protein band intensities were quantified using ImageJ software (National Institutes of Health) (68). Data are presented as means ± standard deviations from three independent experiments.

### Statistics.

All statistical analyses were conducted using GraphPad Prism 8 (GraphPad Software). The statistical significance of viral load, body weight, and cytokine expression over time was determined using two-way analysis of variance (ANOVA). Differences between the wild-type strain and beta variant at specific time points were determined by Student’s *t* test or one-way ANOVA and are presented with horizontal bars between the groups.

### Data availability.

Microarray data sets are available for whole-transcriptome analysis (GEO accession number GSE202998).
