# Supplementary material for: Immunological and Pathological Peculiarity of Severe Acute Respiratory Syndrome Coronavirus 2 Beta Variant
Source: Microbiol Spectr. 2022 Aug 25;10(5):e02371-22. doi: 10.1128/spectrum.02371-22 (PMC9602775; doi:10.1128/spectrum.02371-22)
Supplement: Supplemental file 1 — Fig. S1 to S6. Download spectrum.02371-22-s0001.pdf, PDF file, 1.0 MB [file spectrum.02371-22-s0001.pdf]

Immunological and Pathological Peculiarity of Severe Acute Respiratory Syndrome Coronavirus 2 Beta Variant

Sunhee Lee[1,#*], Gun Young Yoon[1,2*], Su Jin Lee[1*], Young-Chan Kwon[1], Hyun Woo Moon[1], Yu-Jin Kim[1], Haesoo Kim[1], Wooseong Lee[1], Gi Uk Jeong[1], Chonsaeng Kim[1], Kyun-Do Kim[1], Seong-Jun Kim[1†], and Dae-Gyun Ahn[1†]

[1]Center for Convergent Research of Emerging Virus Infection, Korea Research Institute of Chemical Technology, Daejeon 34114, South Korea

[2]Department of Immunology, College of Medicine, Konkuk University, Chungju 27478, South Korea

[#]Current address: Division of Vaccine Clinical Research, Korea National Research Institute of Health, Korea Centers for Disease Control and Prevention, CheongJu 28160, South Korea

*These authors are contributed equally to this work

SUPPLEMENTARY INFORMATION

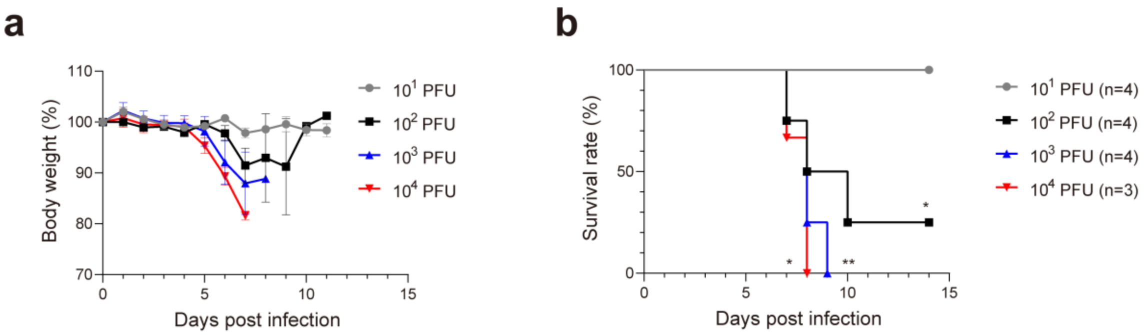

Supplementary Fig. S1. Infection with increasing doses of severe acute respiratory syndrome coronavirus 2 (SARS-CoV-2) beta (β) variant in K18-hACE2 mice. Eight-week-old male K18-hACE2 mice were infected intranasally with 1×10^1 (n=4), 1×10^2 (n=4), 1×10^3 (n=4), and 1×10^4 pfu (n=3) of SARS-CoV-2 β variant. **(a)** Body weight and **(b)** survival rate. Body weight symbols represent mean ± standard error of the mean. *$P < 0.05$ and **$P < 0.01$ (1×10^2, 1×10^3, and 1×10^4 *vs*. 1×10^1 pfu [log-rank (Mantel–Cox) test (survival rate)]).

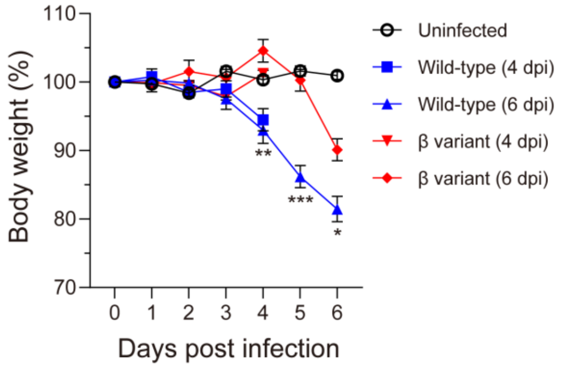

Supplementary Fig. S2. Body weight of K18-hACE2 mice infected with severe acute respiratory syndrome coronavirus 2 (SARS-CoV-2) wild-type (WT) strain or beta (β) variant. Eight-week-old male K18-hACE2 mice (five mice per group) were infected intranasally with 2×10^3 pfu of SARS-CoV-2 WT strain or β variant. Body weight was monitored until sacrifice at 4 and 6 dpi. Body weight symbols represent mean ± standard error of the mean. *$P < 0.05$, **$P < 0.01$, and ***$P < 0.001$ (WT strain *vs.* β variant [two-way analysis of variance with Tukey's multiple comparison test]).

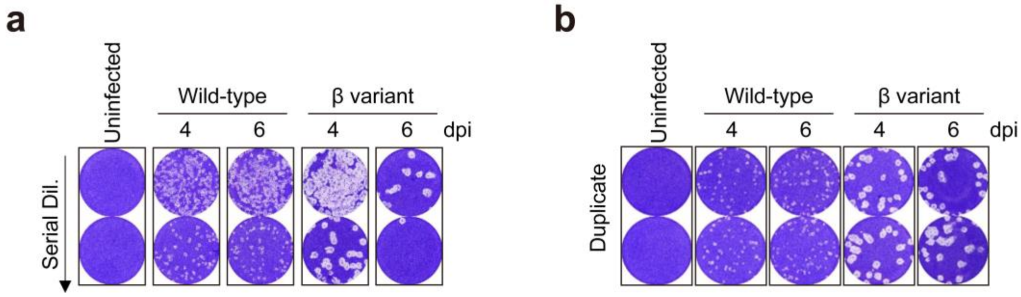

Supplementary Fig. S3. Virus titers in lung homogenates of K18-hACE2 mice infected with severe acute respiratory syndrome coronavirus 2 (SARS-CoV-2) wild-type (WT) strain or beta (β) variant. The number of infectious virus particles was measured by plaque assay in serially diluted (dil.) lung homogenates of K18-hACE2 mice infected with SARS-CoV-2 WT strain or β variant (five mice per group). Plaques were quantified in two independent experiments. Representative plaques **(a)** in the wells of the virus titration plate and **(b)** in the wells of plates with similar numbers of plaques for measuring the mean plaque size.

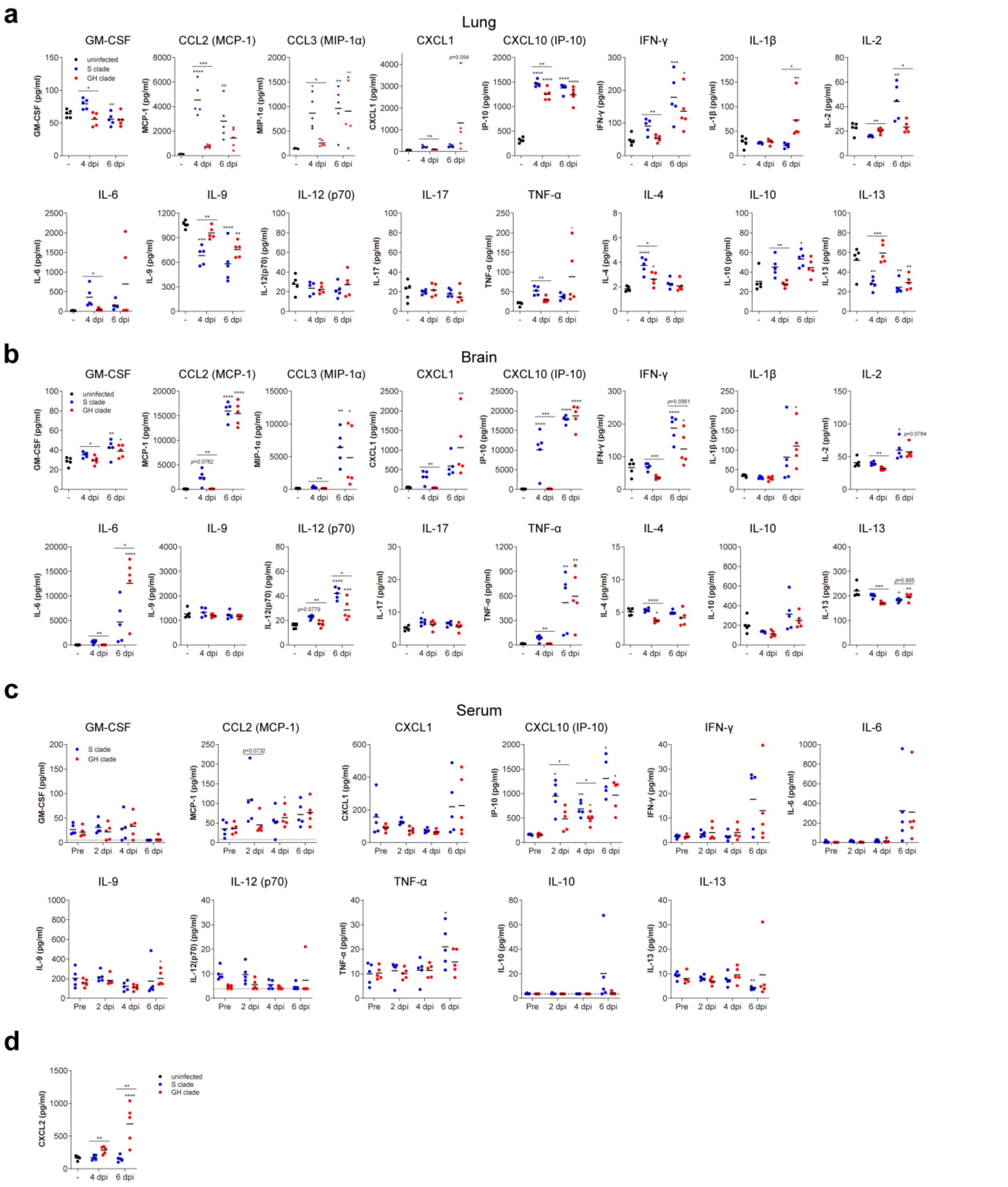

Supplementary Fig. S4. Chemokine and cytokine profiles after severe acute respiratory syndrome coronavirus 2 (SARS-CoV-2) wild-type (WT) or beta (β) variant infection.

Cytokine levels in **(a)** the lungs, **(b)** brain, and **(c)** serum from mice infected with SARS-CoV-2 WT (blue dots) or β variant (red dots) (five mice per group; two independent experiments). Uninfected mice (–) (black dots) were used as controls. For serum analysis, samples collected before infection (pre) were used as controls. Sixteen cytokines were analyzed using a multiplex cytokine assay. $^{*}P < 0.05$, $^{**}P < 0.01$, $^{***}P < 0.001$, and **** $P < 0.0001$ (WT strain or β variant *vs.* mock [two-way analysis of variance with Sidak's multiple comparison test]; WT strain *vs.* β variant [Student's *t*-test]). *P*-values between 0.05 and 0.1 are indicated. Dotted lines represent the limit of detection. **(d)** CXCL2 levels in the lungs measured by enzyme-linked immunosorbent assay.

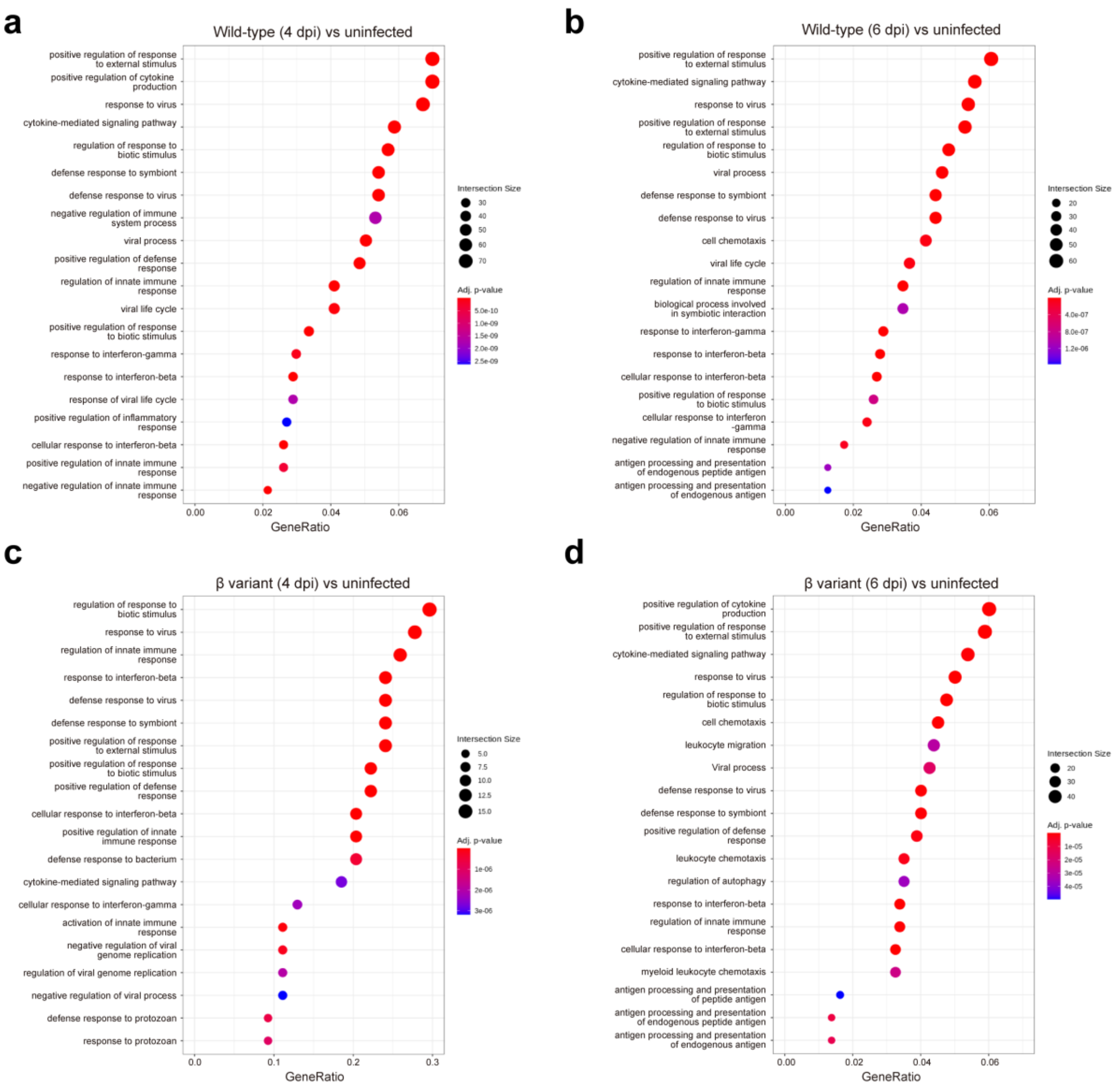

Supplementary Fig. S5. Gene ontology enrichment analysis. The whole transcriptome of lung homogenates from K18-hACE2 mice infected with severe acute respiratory syndrome coronavirus 2 **(a, b)** wild-type strain or **(c, d)** beta variant was analyzed. The top 20 terms are listed according to the size of the intersection and gene ratio. Dot size represents the size of the intersection (number of unique differentially expressed genes [DEGs] that are annotated with the functional category). Dot color represents the false discovery rate-adjusted *P*-value (adj. *P*-value). Gene ratio is the size of the intersection of each term divided by the total number of unique DEGs that are annotated with the functional category.

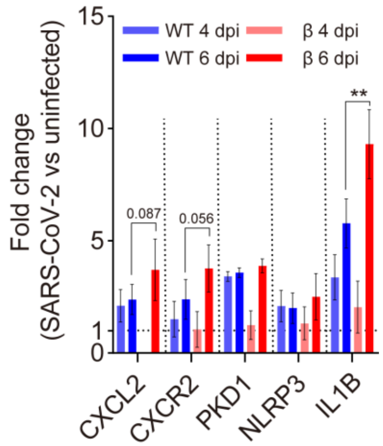

Supplementary Fig. S6. Expression of genes associated with inflammasome activation. Fold-changes of the indicated genes in K18-hACE2 mice infected with severe acute respiratory syndrome coronavirus 2 (SARS-CoV-2) wild-type (WT) strain (blue bars) or beta (β) variant (red bars) compared to uninfected mice (five mice per group). The horizontal dotted line represents a fold-change of 1. Each gene is separated by a vertical dotted line. ***P* < 0.01 (WT strain *vs.* β variant [Student's *t*-test]). *P*-values between 0.05 and 0.1 are indicated.
